# Operational stability study of lactate biosensors: modeling, parameter identification, and stability analysis

**DOI:** 10.3389/fbioe.2024.1385459

**Published:** 2024-07-16

**Authors:** Vasyl Martsenyuk, Oleksandr Soldatkin, Aleksandra Klos-Witkowska, Andriy Sverstiuk, Ksenya Berketa

**Affiliations:** ^1^ Department of Computer Science and Automatics, University of Bielsko-Biala, Bielsko-Biala, Poland; ^2^ Department of Biomolecular Electronics, Institute of Molecular Biology and Genetics of NASU, Kyiv, Ukraine; ^3^ Department of Medical Informatics, Ivan Horbachevsky Ternopil National Medical University of the Ministry of Health of Ukraine, Ternopil, Ukraine; ^4^ Department of Computer Science, Ternopil Ivan Puluj National Technical University, Ternopil, Ukraine

**Keywords:** lactate biosensor, operational stability, Michaelis–Menten kinetics, delay, quasi-polynomial, marginal stability, Poincaré section, bifurcation diagram

## Abstract

**Introduction:**

This paper investigates the operational stability of lactate biosensors, crucial devices in various biomedical and biotechnological applications. We detail the construction of an amperometric transducer tailored for lactate measurement and outline the experimental setup used for empirical validation.

**Methods:**

The modeling framework incorporates Brown and Michaelis–Menten kinetics, integrating both distributed and discrete delays to capture the intricate dynamics of lactate sensing. To ascertain model parameters, we propose a nonlinear optimization method, leveraging initial approximations from the Brown model’s delay values for the subsequent model with discrete delays.

**Results:**

Stability analysis forms a cornerstone of our investigation, centering on linearization around equilibrium states and scrutinizing the real parts of quasi-polynomials. Notably, our findings reveal that the discrete delay model manifests marginal stability, occupying a delicate balance between asymptotic stability and instability. We introduce criteria for verifying marginal stability based on characteristic quasi-polynomial roots, offering practical insights into system behavior.

**Discussion:**

Qalitative examination of the model elucidates the influence of delay on dynamic behavior. We observe a transition from stable focus to limit cycle and period-doubling phenomena with increasing delay values, as evidenced by phase plots and bifurcation diagrams employing Poincaré sections. Additionally, we identify limitations in model applicability, notably the loss of solution positivity with growing delays, underscoring the necessity for cautious interpretation when employing delayed exponential function formulations. This comprehensive study provides valuable insights into the design and operational characteristics of lactate biosensors, offering a robust framework for understanding and optimizing their performance in diverse settings.

## 1 Introduction

### 1.1 Operational stability of biosensor vs. Lyapunov stability of the dynamic model

Operational stability of biosensors means “retention of activity of a protein or enzyme when in use” (Gibson, 1999).

It corresponds mainly with the same notion for dynamic systems from stability theory. Traditionally, enzyme–substrate interaction is simulated with the help of the Michaelis–Menten model, which is a nonlinear dynamic system. On the other hand, previous studies have paid little attention to the qualitative behavior of the model from the viewpoint of its stability. Partially, operational stability deals with the asymptotic nature of the stability notion, whereas biochemists conduct experiments during “finite time.”

In turn, the stability theory of dynamic systems, so-called Lyapunov stability, offers powerful tools for enhancing biochemical reactions modeling and saving the qualitative behavior of the systems. Moreover, each model is based on a series of assumptions about biochemical interactions, which allows us to check the validity of phenomena that cannot be verified experimentally, for example, mass action law with the distributed or discrete delays, which will be presented later.

### 1.2 Background for lactate measurement

**FIGURE 1:**
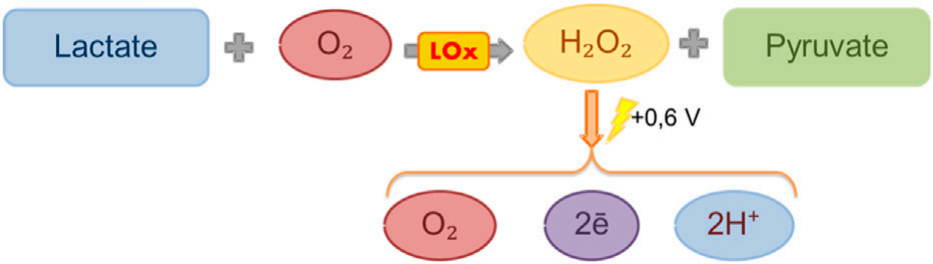
Scheme of reactions that underlie the functioning of a biosensor.

Lactate characteristics: Lactate is an anion of lactic acid and is the final metabolite of the anaerobic breakdown of glucose. It is formed from pyruvate during the processes of glycolysis in the absence of oxygen (Figure 1) (Rabinowitz and Enerbäck, 2020) and is an important substance used in medicine as a marker of hypoxia and a number of other disorders, including diabetes and liver and kidney disorders (Pundir et al., 2016).

Lactate is also actively used in the food industry as an emulsifier, thickener, and acidity regulator. Various salts of lactic acid in the international classification of food additives are numbered E325, E326, E327, E328, and E329. Magnesium lactate is sometimes classified as an antioxidant (Standards, 2021). In addition, lactate can act as an indicator of the activity of bacteria during the fermentation process (Rawoof et al., 2020). It can indicate the freshness and quality of some products—wine (Gamella et al., 2010), juices (Trifirò et al., 1997), etc.

The concentration of lactate in products increases as they spoil due to changes in organoleptic properties. Due to the wide use of lactate as a marker of many processes in medicine and industry, accurate and effective methods of its diagnosis are necessary.

Lactate measurement methods: Traditionally, lactate is measured by colorimetry (Suman et al., 2005; Pundir et al., 2016), spectrophotometry (Pundir et al., 2016), fluorometry (Xue and Yeung, 1994), liquid chromatography (Biagi et al., 2012), and nuclear magnetic resonance (Hu et al., 2012; Park et al., 2015). These methods are certainly effective but have several disadvantages, such as high cost, length, and complexity of the analysis. Some of these methods also require complex preliminary sample preparation.

### 1.3 State-of-the-art lactate measurement with the help of biosensors

Biosensors offer effective and sensitive detection methods that can be used in medical institutions to measure the level of lactate in blood, sweat, and other biological fluids. In addition, new methods of lactate detection can be useful for enterprises that must control the processes of manufacturing food products and pharmaceuticals (Rattu et al., 2020).

Classification of lactate biosensors from the viewpoint of a bioselective element: A number of biosensor developments aimed at determining the concentration of lactate are known to be in development. Known biosensors can be divided according to the type of bioselective element: sensors based on lactate oxidase (LOx) and sensors based on lactate dehydrogenase (LHD) (Rassaei et al., 2013). In both cases, the substrate and product of the enzymatic reaction are lactate and pyruvate, respectively.

Basic reactions for lactate biosensors: There is a significant difference in these two reactions. For the LHD reaction, NAD+ is needed as a proton carrier in the reaction of dehydrogenation of lactate to pyruvate (Chatterjee et al., 2023). The lactate oxidase reaction can be much easier because, in it, the role of the proton acceptor is played by oxygen; that is, the use of this enzyme in biosensors does not require additional reagents (Lockridge et al., 1972).

Other variants of biosensors are based on a mixture of LHD and LOx. This configuration of the bioselective element, according to the results obtained by Chaubey et al. (2000), allows measuring lactate at lower concentrations than mono-enzyme biosensors, but this makes the analysis more expensive and is characterized by the complexity of manufacturing.

Classification of lactate biosensors from the viewpoint of the transducer: The main biosensor measurement methods used for lactate analysis are optical—electrochemiluminescence or fluorescence (Pundir and Narwal, 2017) and electrochemical—amperometric, potentiometric, or, less often, conductometric or impedimetric (Rathee et al., 2016). Moreover, various methods of improving the main characteristics of sensors have been applied to known biosensor systems—nanoparticles (Nesakumar et al., 2013; Azzouzi et al., 2015), other nanomaterials (Cui et al., 2007), complex, multi-stage methods of enzyme immobilization (Parra et al., 2006), and multi-enzyme membranes.

Although amperometric (Romero et al., 2010) and potentiometric (Mengarda et al., 2019) biosensors are usually monoenzymatic, that is, based on LHD or Lx, conductometric (Nguyen-Boisse et al., 2013) and impedimetric (Chan et al., 2017) biosensors usually use two-enzyme bioselective elements, for example, based on a mixture of lactate oxidase and peroxidase (Nguyen-Boisse et al., 2013) or a mixture of lactate dehydrogenase and pyruvate oxidase (Chan et al., 2017).

Motivation for lactate biosensor design: Therefore, in connection with the variety of works on the development of biosensor systems for measuring lactate and the prospect of its wide use in various areas of human life, we have concluded that the development of new biosensor methods for determining lactate is an urgent need.

Most existing biosensors are currently not ready for wide implementation and commercialization due to various limitations, such as insufficient sensitivity, selectivity, stability with respect to possible inferents, or too narrow a range of biosensor operation.

### 1.4 Michaelis–Menten model for enzyme kinetics

According to the model, an enzyme E combines with a substrate S to form an enzyme–substrate complex ES, characterized by a rate constant *k*_1_. The resulting complex can dissociate into E and S (with a rate constant of *k*_−1_) or transform into a product P with a rate constant of *k*_2_ (Berg et al., 2002).

The speed of the enzyme process is dependent on the ease of formation of the complex of the enzyme with the substrate. For low substrate concentrations, the reaction rate is proportional to the substrate concentration, while at higher concentrations, it tends toward a maximum value and becomes independent of substrate concentration. The general dependence of the rate of an enzyme reaction on substrate concentration is described by an equation called the Michaelis–Menten equation (Eq. 1):

$$v=\frac{V_{\max}n_{S}}{K_{M}+n_{S}}.$$ (1)

Here, *v* is the rate of reaction (mol/s), *V*_max_ is the maximum reaction rate (mol/s), *n*_*S*_ is substrate concentration (mol/dm^3^), and *K*_*M*_ is the Michaelis–Menten constant (mol/dm^3^).

The Michaelis–Menten constant from Eq. 1 is an enzyme-specific quantity, dependent on substrate, temperature, and pH and independent of enzyme concentration. This constant is a measure of the affinity of the enzyme for the substrate. The lower the value of the constant, the higher the affinity of the enzyme for the substrate (Berg et al., 2002; Radomska, 2016).

### 1.5 Stability research on enzyme kinetics

Models of enzyme kinetics are based on compartmental systems, which are dynamic systems characterized by a network of interconnected nodes, each representing a reservoir or compartment where resources are stored (Blanchini et al., 2023). The system’s behavior is governed by the movement of resources, depicted as flows traveling along the edges connecting these compartments. Compartment-based dynamic systems serve as invaluable models across various disciplines, including physiologically based pharmacokinetics (Martsenyuk et al., 2012; Thompson and Beard, 2012), mathematical epidemiology (Reyné et al., 2022; Martsenyuk et al., 2021a; b), enzyme kinetics (Keener and Sneyd, 2009; Craciun et al., 2020; Martsenyuk et al., 2022), demography (Navarro Valencia et al., 2023), and ecology (Krishna et al., 2024). Stability analysis plays a pivotal role in understanding the behavior of these systems under different conditions (Blanchini et al., 2023). In recent years, researchers have made significant strides in advancing stability research methodologies, particularly in the context of compartmental systems with delay (Martsenyuk et al., 2013; Martsenyuk and Gandzyuk, 2013).

One common approach to stability analysis is linearization, which involves approximating nonlinear systems around equilibrium points. This technique has been extensively utilized in physiologically based pharmacokinetic models to assess the stability of drug distribution processes within the body (Martsenyuk et al., 2012). Lyapunov functions represent another powerful tool for stability analysis, offering a rigorous mathematical framework to prove the stability properties of compartment-based systems. In mathematical epidemiology, Lyapunov functions have been employed to establish global stability of disease-free and endemic equilibria in compartmental models of infectious diseases (Martsenyuk et al., 2021a).

### 1.6 Brief description of the work

Section 2 describes the materials, including the experiment and models used. The methods presented are related to parameter identification and stability research. Section 3 shows the results concerning the parameter identification for models using gamma-distributed delay and with two discrete delays. The qualitative analysis includes the existence and positiveness of the solutions, equilibrium states, marginal stability, and numerical research with the help of Poincaré sections. In Section 4, we discuss the results obtained and open problems.

The *objective* of the work is to offer the flowchart of the lactate biosensor design, including modeling, parameter identification, and stability analysis.

## 2 Materials and methods

### 2.1 Chemical compounds

The enzyme lactate oxidase obtained from *Aerococcus viridans* with an activity of 100 units (Sigma, United States) was used to create the biosensor. Bovine serum albumin (fraction V) (BSA) and a 25% aqueous solution of glutaraldehyde (GA), glycerol, and KCl were obtained from Sigma-Aldrich (United States). Stock 500 mM sodium L-lactate solution (Sigma-Aldrich, Switzerland) was used as a substrate. HEPES obtained from Sigma-Aldrich (United States) was used to prepare the buffer solution. Other inorganic compounds used in the work were of domestic production and had a purity level of “h.p.” and “p.d.a.”

### 2.2 Lactate biosensor design

**FIGURE 2:**
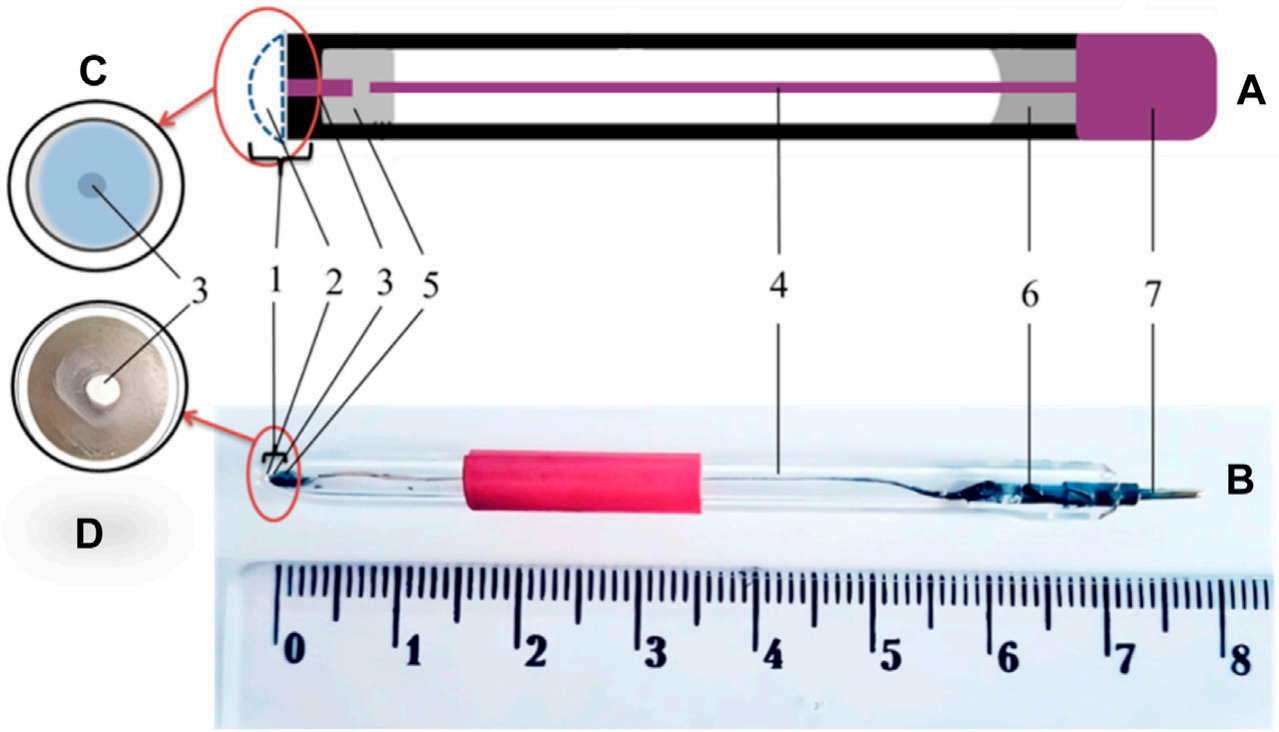
General scheme of amperometric transducers. **(A)** Schematic view of the amperometric transducer. **(B)** Photograph of an amperometric transducer. **(C)** Scheme of the sensitive area of the transducer. **(D)** Photograph of the sensitive area of a transducer. 1–sensitive area, 2 –enzyme membrane, 3–platinum wire, 4–inner conductor (silver wire), electrical connection using 5–low-melting Wood’s alloy, 6–epoxy resin, and 7–contact area.

The general scheme of amperometric transducers is shown in Figure 2. Platinum disc electrodes were used as amperometric transducers, manufactured in the laboratory of the Department of Biomolecular Electronics of the Institute of Biomolecular Biology and Geosciences using the following technology: a piece of platinum electrode with a diameter of 0.5 mm and a length of 3 mm was placed in a capillary tube with an outer diameter of 3.5 mm, and then the narrowed end of the capillary tube was sealed in a torch flame. The electrical connection between the platinum and the silver wire conductor was made by low-temperature soldering using Wood’s alloy. The open end of the capillary was filled with epoxy resin, with part of the conductor inside the capillary and part outside. A copper contact was soldered to the conductor to connect the transducer to the measuring unit.

**FIGURE 3:**
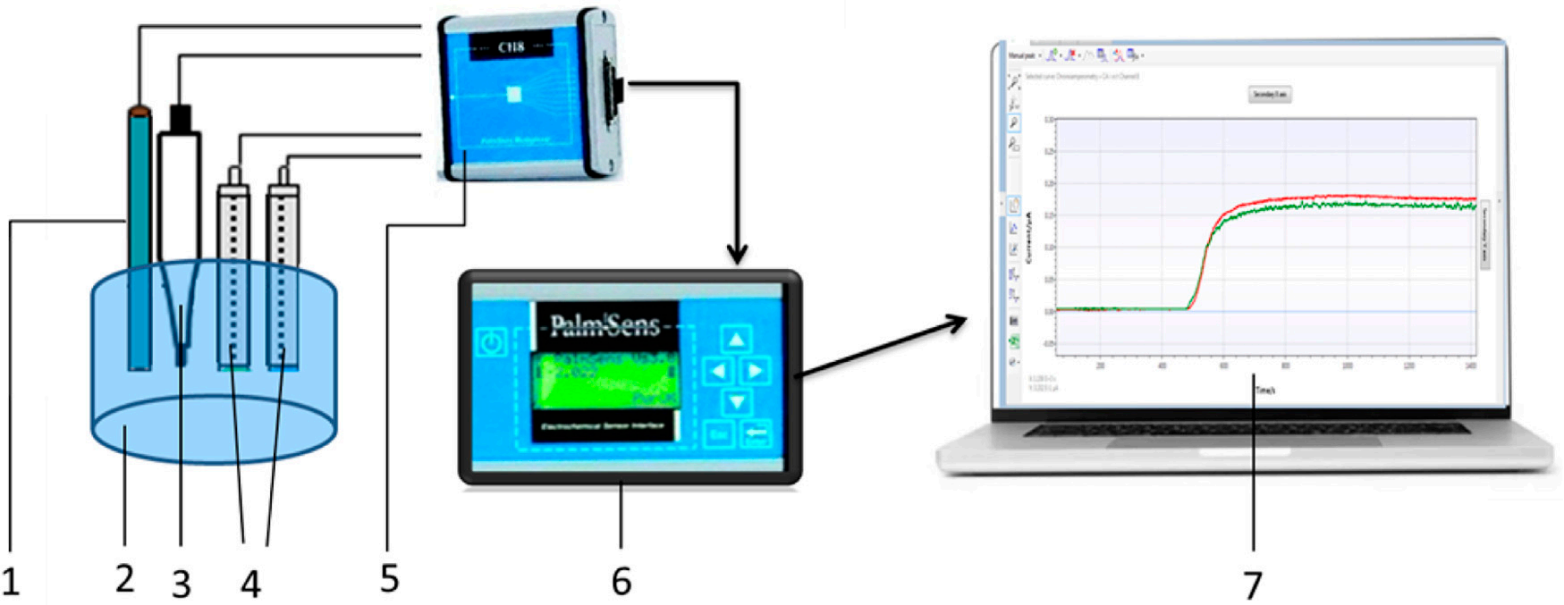
Scheme of the amperometric measurement setup. 1–auxiliary electrode, 2–measurement cell, 3–reference electrode, 4–working electrodes, 5–multiplexer, 6–potentiostat, and 7–PSTrace, measurement software for the potentiostat.

Amperometric measurements setup is shown in Figure 3. The PalmSens potentiostat (Palm Instruments BV, the Netherlands) was connected to an auxiliary platinum electrode, a silver chloride (Ag/AgCl) reference electrode, and working electrodes based on platinum disc electrodes. The potentiostat was connected to an 8-channel device (CH-8 multiplexer, Palm Instruments BV, the Netherlands), which allowed it to receive signals simultaneously from several working electrodes or biosensors (up to eight simultaneously). The distance between the auxiliary platinum electrode and all working biosensors during the measurement was the same (approximately 5 mm).

Preparation of bioselective membranes: Bioselective membranes were prepared by immobilizing proteins on the surface of a platinum disc electrode by covalent cross-linking of the enzyme in a bovine serum albumin matrix using glutaraldehyde as a cross-linking agent. The enzyme gel containing 5% lactotoxidase, 5% BSA, and 10% glycerol was mixed with a 1% glutaraldehyde solution in a 1:1 ratio, after which the mixture was applied to the sensitive area of the platinum disc electrode, and the membrane was air-dried for 20 min. After immobilization, the residual glutaraldehyde and unbound membrane components were washed off the membranes in a buffer solution for 5 min with constant stirring, changing the buffer several times.

### 2.3 Description of amperometric measurements

The measurement was carried out in an open measuring cell with a volume of 2 mL under constant stirring at room temperature. A fixed potential of +0.6 V was applied to the electrodes relative to the chloride silver reference electrode. The working buffer was 25 mM HEPES with pH 7.4. The required concentration of the substrate in the cell was set by adding aliquots of the standard solution, 500 mM sodium L-lactate, to the buffer.

The duration of one response (from the addition of the substrate to the signal output to the baseline) was approximately 4–5 min; between responses, the substrate was washed off the biosensor for 5 min, changing the buffer in the measuring cell several times. All measurements were performed in at least three replicates.

### 2.4 Models used

#### 2.4.1 Model with continuously distributed delays

An application of delayed mass action law to enzyme kinetics was inspired by Brown’s model, formulated by Brown (1902), where complex C has a lifetime *τ* before being decayed. We called the reaction scheme

$$\text{E}\left(\text{t}\right)+\text{S}\left(\text{t}\right)\overset{{\text{k}}_{\text{d}}}{\to }\text{E}\left(\text{t}+\tau \right)+\text{P}\left(\text{t}+\tau \right)$$

an irreversible one-complex Brown’s (IR1CB) mechanism. In Martsenyuk et al. (2022), we offered the following model based on continuously distributed delays:

$$\begin{array}{ll} \frac{dn_{S}\left(t\right)}{dt} & =-k_{d}n_{E}\left(t\right)n_{S}\left(t\right),\\ \frac{dn_{E}\left(t\right)}{dt} & =-k_{d}n_{E}\left(t\right)n_{S}\left(t\right)+k_{d}\int_{-\tau_{M}}^{0}f\left(s\right)n_{E}\left(t+s\right)n_{S}\left(t+s\right)ds,\\ \frac{dn_{P}\left(t\right)}{dt} & =k_{d}\int_{-\tau_{M}}^{0}f\left(s\right)n_{E}\left(t+s\right)n_{S}\left(t+s\right)ds, \end{array}$$ (2)

where for confidence level *c* ∈ (0, 1), we set $\tau_{M}≔\tau_{\min}+\frac{m+1}{a}+\sqrt{\frac{\left(m+1\right)}{a^{2}\left(1-c\right)}}$ , *f*(*s*) is the density function of the delay distribution, which was designed in the form of a gamma distribution:

$$f\left(a,m,\tau_{\min},s\right)≔\left\{\begin{array}{cc} 0\; & s\leq \tau_{\min},\\ \frac{a^{m+1}}{\Gamma \left(m+1\right)}{\left(s-\tau_{\min}\right)}^{m}e^{-a\left(s-\tau_{\min}\right)}\; & s>\tau_{\min}, \end{array}\right.$$ (3)

where *a*, *m*, *τ*_min_ ≥ 0 are the parameters that determine the corresponding probability density function. Their roles and the ways of estimating were well-studied in Martsenyuk et al. (2022). The basic idea of Brown’s model shown in Model (2) does not include complex C directly but involves the model time *τ* required for complex forming–destroying. The model parameters were well-studied by Martsenyuk et al. (2022) and can be used as an initial approximation for the complex-based model in the next subsection.

#### 2.4.2 Models with two discrete delays

The model extends the well-known irreversible one-complex Michaelis–Menten (IR1CMM) mechanism (Keener and Sneyd, 2009) (Section 1.4)

$$E+S\overset{k_{1}}{\underset{k_{-1}}{⇋}}C\overset{k_{2}}{\to }E+P$$

by adding the time durations *τ*_1_ and *τ*_2_ required for the entire fulfillment of the forward reactions. Mathematically, it corresponds to the time delays within dynamic systems. Hence, we consider the following set of elementary reactions:

$$\begin{array}{ll} & \text{S}+\text{E}\overset{{\text{k}}_{1},\tau_{1}}{\to }\text{C}\\ & \text{C}\overset{{\text{k}}_{-1}}{\to }\text{S}+\text{E}\\ & \text{C}\overset{{\text{k}}_{2},\tau_{2}}{\to }\text{E}+\text{P}, \end{array}$$

which we call the irreversible one-complex with two delays Michaelis–Menthen (IR1C2DMM) mechanism. Based on the general approach described by Craciun et al. (2020), it yields the delayed model

$$\begin{array}{ll} & \frac{dn_{S}}{dt}=k_{-1}n_{C}-k_{1}n_{S}\left(t-\tau_{1}\right)n_{E}\left(t-\tau_{1}\right)\\ & \frac{dn_{E}}{dt}=k_{-1}n_{C}+k_{2}n_{C}\left(t-\tau_{2}\right)-k_{1}n_{S}\left(t-\tau_{1}\right)n_{E}\left(t-\tau_{1}\right)\\ & \frac{dn_{C}}{dt}=k_{1}n_{S}\left(t-\tau_{1}\right)n_{E}\left(t-\tau_{1}\right)-k_{2}n_{C}\left(t-\tau_{2}\right)-k_{-1}n_{C}\\ & \frac{dn_{P}}{dt}=k_{2}n_{C}\left(t-\tau_{2}\right). \end{array}$$ (4)

For the solutions of Eq. 4, elements of which are the vector functions *n*_*S*_, *n*_*E*_, *n*_*C*_, *n*_*P*_ ∈ **C** ^1^ ([−*τ*_max_], 0], **R** ^4^), we consider the following initial conditions:

$$\begin{array}{ll} & n_{S}\left(t\right)={\hat{n}}_{S}\left(t\right)\geq 0,n_{E}\left(t\right)={\hat{n}}_{E}\left(t\right)\geq 0,n_{C}\left(t\right)={\hat{n}}_{C}\left(t\right)\geq 0,n_{P}\left(t\right)={\hat{n}}_{P}\left(t\right)\geq 0,t\in \left[-\tau_{\max},0\right),n_{S}\left(0\right)={\hat{n}}_{S}^{0}>0,n_{E}\left(t\right)={\hat{n}}_{E}^{0}>0,n_{C}\left(t\right)={\hat{n}}_{C}^{0}>0,n_{P}\left(t\right)={\hat{n}}_{P}^{0}>0. \end{array}$$ (5)

The value of *n*_*P*_(*t*) can be found by direct integration, namely,

$$n_{P}\left(t\right)=n_{P}\left(0\right)+k_{2}\int_{0}^{t}n_{C}\left(s-\tau_{2}\right)ds,\;t>0.$$ (6)

### 2.5 Methods

#### 2.5.1 Parameter identification

As a result of the amperometric measurements described in Section 2, we obtain responses in the form of current *I*(*t*). We propose to use the relation of the current *I*(*t*) with *n*_*P*_(*t*) as

$$I\left(t\right)≔\Lambda_{m}^{0}n_{P}\left(t\right)-K{\left(n_{P}\left(t\right)\right)}^{3/2},\;t>0.$$ (7)

In Martsenyuk et al. (2022), this relationship was evidenced for the specific conductance *κ*(*t*) of conductometric biosensors. Mathematical modeling of conductometric biosensors in terms of conductivity is presented in detail by Zouaoui et al. (2022). Provided fixed potential, we assume the linear dependence of the specific conductance and the current. So, we follow Eq. 7. Numerical modeling regarding amperometric biosensors is displayed by Simelevicius et al. (2012) and Hashem Zadeh et al. (2020).

In the following, we will denote product concentrations obtained as the solutions of the models as *n*_*P*,*pred*_(*t*). In turn, the corresponding values of the current due to Eq. 7 will be *I*_*pred*_(*t*). On the other hand, let *I*_exp_(*t*) be the values of responses received as a result of the experiments.

The proposed parameter identification uses the currents *I*_exp*,j*_ (*t*_*i*_) and *I*_*pred,j*_ (*t*_*i*_), $j=\bar{1,m}$ , experimentally and numerically obtained at the time instances *t*_*i*_, $i=\bar{1,N}$ ^1^ for given initial substrate concentrations *n*_*S*_ (0) = *n*_*S*,*j*_.

Our goal is to estimate the parameters $\Pi_{\text{IR}1\text{CB}}=\left\{k_{d},a,m,\tau_{\min},\Lambda_{m}^{0},K\right\}\in R_{+}^{6}$ , $\Pi_{\text{IR}1\text{C}2\text{DMM}}=\left\{k_{1},k_{2},k_{-1},\tau_{1},\tau_{2},\Lambda_{m}^{0},K\right\}\in R_{+}^{7}$ when applying Model (2) or (4), respectively.

We will estimate the parameters Π of the models solving the problems of optimization:

$$\left.\begin{array}{cc} minimize & J\left(\Pi \right)\\ subjectto & g_{i}\left(\Pi \right)\geq \Theta ,\;i=1,2. \end{array}\right\}$$ (8)

Here,

$$J\left(\Pi \right)≔{\left(\overset{m}{\underset{j=1}{\sum }}\left(\overset{N}{\underset{i=1}{\sum }}\left({\left(I_{\mathrm{exp,j}}\left(t_{i}\right)-I_{\mathrm{pred,j}}\left(t_{i}\right)\right)}^{2}\right)\right)\right)}^{1/2}$$ (9)

is the target function, and

$$\begin{array}{cc} g_{1}\left(\Pi \right)= & \Pi -\Pi_{\mathrm{lower}}\geq \Theta ,\\ g_{2}\left(\Pi \right)= & \Pi_{\mathrm{upper}}-\Pi \geq \Theta , \end{array}$$ (10)

are inequality constraints, where Θ is a null vector, and Π_*lower*_ and Π_*upper*_ are lower and upper bounds for the parameter values of corresponding dimensions.

An algorithm for the estimation of Π_IR1CB_ was described in detail by Martsenyuk et al. (2022). Here, we also apply it for the estimation of Π_IR1C2DMM_. Moreover, the values of *a*, *m*, and *τ*_min,_ obtained as a result of the parameter identification in Eq, 2, will be used in order to set the initial estimate for *τ*_1_ and *τ*_2_ when estimating Eq. 4. Note that the mean value of time delay for Model (2) can be calculated as

$$E\left(\tau \right)≔\tau_{\min}+\frac{m+1}{a}.$$

So, we set the initial values of delays from Eq. 4 such that $\tau_{1}+\tau_{2}\approx E\left(\tau \right)$ .

#### 2.5.2 Stability research using linearization technique

We will conduct research on local stability using the linearization technique. In this case, stability conditions are constructed based on a characteristic quasi-polynomial. The signs of the real parts of its roots are decisive for making conclusions about stability.

Namely, if all roots lie on the open left-half plane, then the equilibrium is locally asymptotically stable. If some of the roots have positive real parts, then the equilibrium is unstable. We focus our attention on the case when the roots lie on a closed left-half plane; that is, we have some simple roots lying on an imaginary axis. Such a situation is known as marginal stability. We distinguish this situation from the case of the pair of purely imaginary roots corresponding to periodic solutions.

When investigating the characteristic quasi-polynomial, a Padé approximant will be used in the form

$$e^{-\lambda \tau }\approx \frac{1-\frac{\lambda \tau }{2}}{1+\frac{\lambda \tau }{2}},$$ (11)

allowing us to approximate the characteristic quasi-polynomial with the help of rational functions (Baker and Graves-Morris, 1996).

#### 2.5.3 Bifurcation plots using Poincaré section

We use the Poincaré section technique to study the qualitative behavior of the models developed, which was primarily applied to the compartmental model by Martsenyuk et al. (2021a).

To begin, we gain a thorough understanding of Model (4), including its equations and parameters. We select parameters *τ*_1_ and *τ*_2_ to vary and the variables *n*_*S*_, *n*_*E*_, and *n*_*C*_ to focus on. Then, we simulate the system across different parameter values, generating time series data for the chosen variables.

Poincaré sections are constructed by intersecting the trajectory of the system with a defined plane *n*_*E*_ = *d* in the phase space, where *d* = (min_*t*_ *n*_*E*_(*t*) + max_*t*_ *n*_*E*_(*t*))/2. This section is determined by specific criteria; namely, we choose such points $\left(n_{S}^{⋆},d,n_{C}^{⋆}\right)$ such that crossing a plane *n*_*E*_ = *d* will happen at sequential time instances *t* ^⋆^ + *T*, *t* ^⋆^ + 2*T*, *t* ^⋆^ + 3*T*, … , where T is a period value; that is, $n_{S}^{⋆}=n_{S}\left(t^{⋆}\right)=n_{S}\left(t^{⋆}+T\right)=n_{S}\left(t^{⋆}+2T\right)=\ldots \;$ . We plot the sampled points in an (*n*_*S*_, *n*_*E*_)-space to visualize the Poincaré sections.

Repeating this process for various parameter values (*τ*_1_, *τ*_2_), we observe how the Poincaré sections change. Analyzing the patterns in the sections, we understand the system’s behavior, including the emergence of periodic orbits, chaotic dynamics, or transitions between different states.

Finally, we summarize the results by constructing a bifurcation plot combined with the corresponding *n*_*S*_, *n*_*E*_ and (*n*_*S*_, *n*_*P*_) phase plots, showing how features of the Poincaré sections vary with parameter values. This comprehensive approach allows us to systematically explore the system’s behavior and gain insights into its dynamics.

## 3 Results

### 3.1 Parameter identification for a model using a gamma-distributed delay

The parameter identification technique mentioned in Section 2.5.1 was used for Model (2). The training data correspond to a set of time series of currents corresponding to given initial substrate concentrations *n*_*S*_(0) equal to 0.1 mM, 0.5 mM, 1.0 mM, and 2.5 mM sequentially.

The goal is to estimate the parameters $\Pi_{\text{IR}1\text{CB}}=\left\{k_{d},a,m,\tau_{\min},\Lambda_{m}^{0},K\right\}\in R_{+}^{6}$ . The initial values for the estimation were chosen as Π_*init*_ (see Table 1). The lower and upper bounds for Π_IR1CB_ are shown in Table 1 as Π_*lower*_ and Π_*upper*_, respectively.

**TABLE 1:** Parameter identification for Model (2).

	*k*_*d*_	*a*	*m*	*τ*_min_	*K*	$\Lambda_{m}^{0}$
Π_*lower*_	1.e^−10^	1.e^−10^	1.e^−10^	1.e^−10^	1.e^−10^	1.e^−10^
Π_*upper*_	1	1,000	1,000	1,000	1.e^6^	1.e^6^
Π_*init*_	0.1	1	20	5	1.e^−10^	0.5
Π_*opt*_	0.09647381	0.9180534	16.2354	4.607475	1e^−10^	0.06818154

**FIGURE 4:**
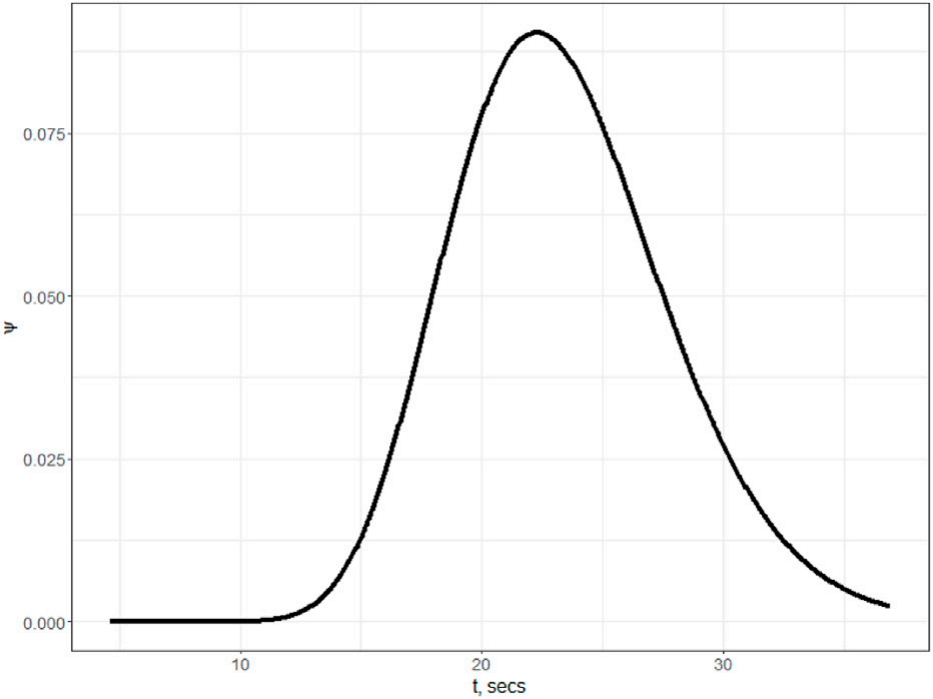
Density function given in 3 for the distributed delay *τ* value from Model Eq. (2).

The most valuable information obtained from Model (2) is the density of the distributed delay distribution (see Figure 4), which will be used in further estimations.

**FIGURE 5:**
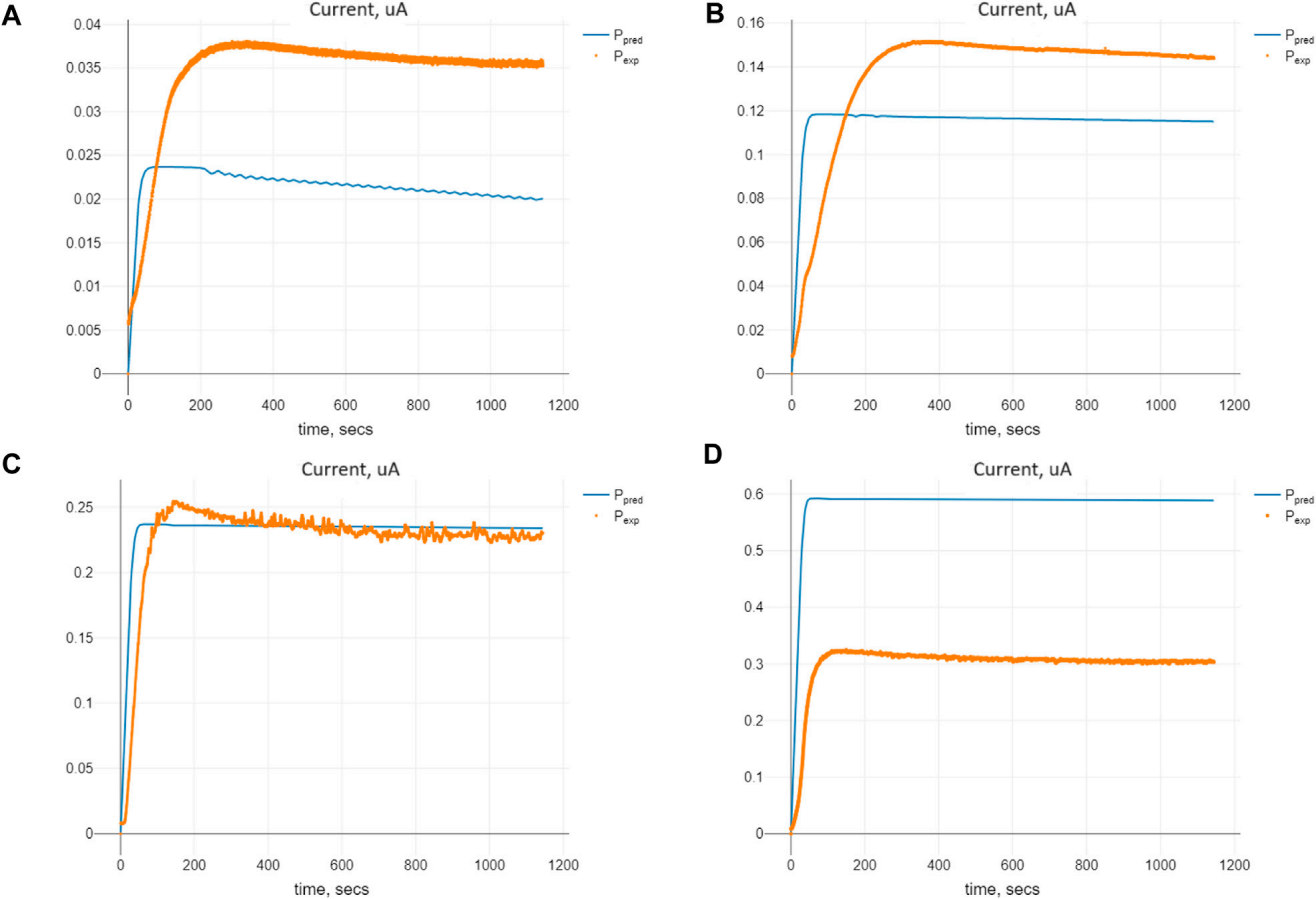
Comparison of the predicted and expected currents for Model (2) at the optimal values of the parameters Π_*opt*_ corresponding to given initial substrate concentrations *n*_*S*_ (0): **(A)** 0.1 mM, **(B)** 0.5 mM, **(C)** 1.0 mM, and **(D)** 2.5 mM.

The comparison of predicted and expected values of the currents for the optimal set of the parameters Π_*opt*_ is shown in Figure 5. We see that the parameter values, being optimal concerning the cost criterion (8), enable us to find the solution of Model (2) closest to the expected currents for the initial substrate concentration of 1.0 mM. For the smaller initial values of the substrate, we have predicted values smaller than the expected ones, whereas for those bigger than 1.0 mM, the predicted values are larger than the experimental ones. The explanation of such an effect lies in the special kind of stability of the model known as marginal stability, which will be evidenced further.

### 3.2 Parameter identification for a model with two discrete delays

Model (4) requires the estimating the parameters $\Pi_{\text{IR}1\text{C}2\text{DMM}}=\left\{k_{1},k_{2},k_{-1},\tau_{1},\tau_{2},\Lambda_{m}^{0},K\right\}\in R_{+}^{7}$ . They were obtained as a result of the solution of the optimization problem shown in Eqs 8–10. The training data described in Section 3.1 were used.

The initial values for the estimation were chosen as Π_*init*_ (see Table 2). The lower and upper bounds for Π_IR1C2DMM_ are shown in Table 2 as Π_*lower*_ and Π_*upper*_, respectively.

**TABLE 2:** Parameter identification for Model (4).

	*k*_1_	*k*_−1_	*k*_2_	*τ*_1_	*K*	$\Lambda_{m}^{0}$	*τ*_2_
Π_*lower*_	1.e^−10^	1.e^−10^	1.e^−10^	1.e^−10^	1.e^−10^	1.e^−10^	0
Π_*upper*_	1	1,000	1,000	1,000	1.e^6^	1.e^6^	1,000
Π_*init*_	0.1	0.1	0.1	15	0.5	0.0	7
Π_*opt*_	0.08717502	0.1048326	0.1220813	15.69849	1.171464e^-05^	0.2323843	5.357829

**FIGURE 6:**
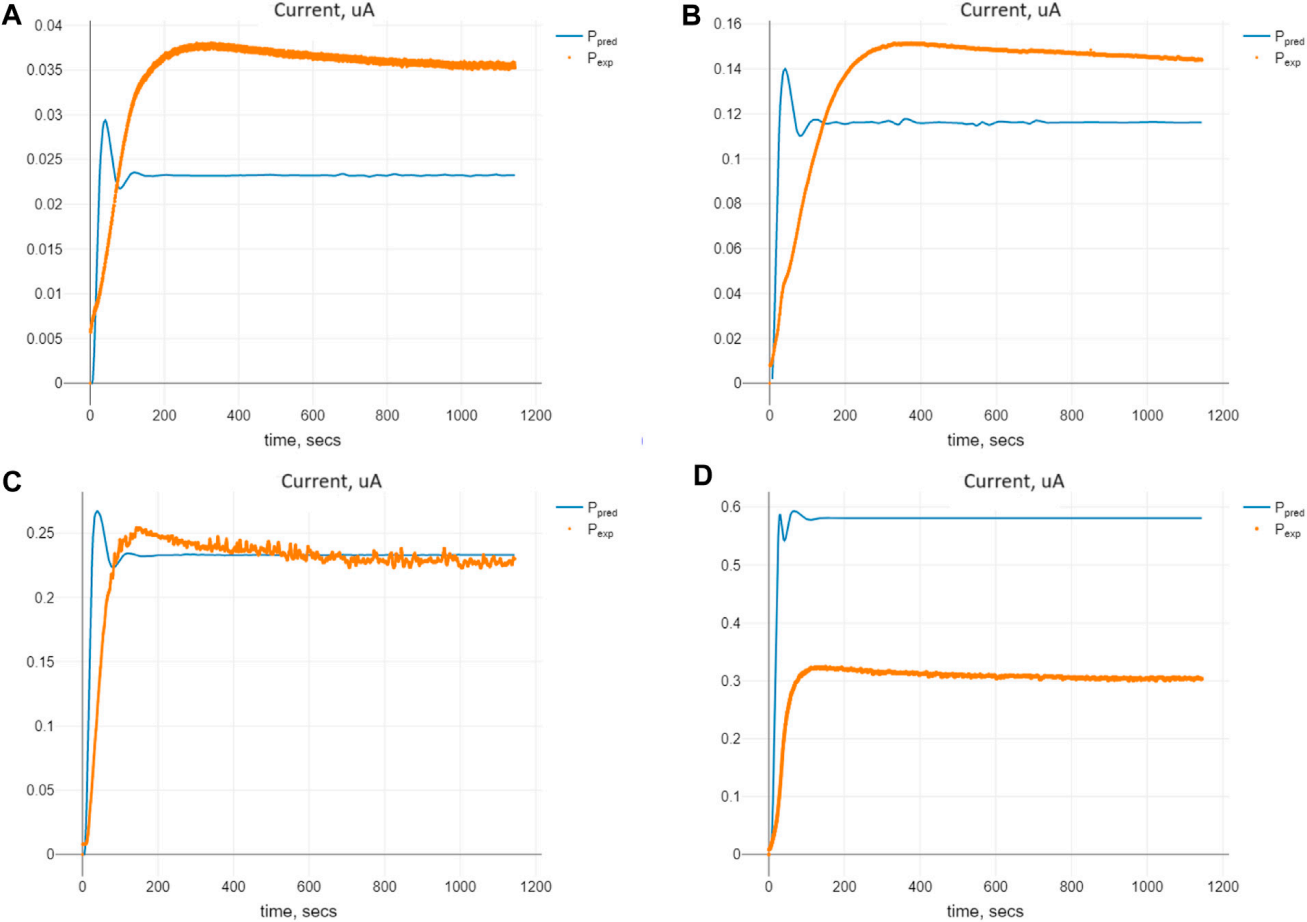
Comparison of the predicted and expected currents for Model (4) at the optimal values of the parameters Π_*opt*_ corresponding to the given initial substrate concentrations *n*_*S*_ (0): **(A)** 0.1 mM, **(B)** 0.5 mM, **(C)** 1.0 mM, and **(D)** 2.5 mM.

The comparison of predicted and expected values of the currents for the optimal set of the parameters Π_*opt*_ for Model (4) is shown in Figure 6. We see similar tendencies of expected and predicted values as we did for Model (2), with gamma-distributed delays.

### 3.3 Qualitative analysis

#### 3.3.1 Existence and positiveness of the solutions

Given ${\hat{n}}_{S}\left(t\right),{\hat{n}}_{E}\left(t\right),{\hat{n}}_{C}\left(t\right),{\hat{n}}_{P}\left(t\right)\in C^{+}\left[-\tau_{\max},0\right]$ , because the right-hand sides of Eq. 4 imply the Lipschitz condition, there exists a unique trajectory of Eq. 4 starting from Eq. 5 (Hale and Lunel, 2013).

Henceforth, we will focus our attention on the positiveness of the solution of the system shown in Eq. 4.

The positiveness of *n*_*P*_(*t*) follows directly from the positiveness of *n*_*C*_(*t*). So, we prove the positiveness of *n*_*S*_(*t*), *n*_*E*_(*t*), and *n*_*C*_(*t*) by contradiction.

##### 3.3.1.1 Case without delays

First, we demonstrate the positiveness for Model (4) without delays, that is, if *τ*_1_ = *τ*_2_ = 0

$$\begin{array}{ll} & \frac{dn_{S}}{dt}=k_{-1}n_{C}-k_{1}n_{S}n_{E}\\ & \frac{dn_{E}}{dt}=k_{-1}n_{C}+k_{2}n_{C}-k_{1}n_{S}n_{E}\\ & \frac{dn_{C}}{dt}=k_{1}n_{S}n_{E}-k_{2}n_{C}-k_{-1}n_{C}\\ & \frac{dn_{P}}{dt}=k_{2}n_{C}. \end{array}$$ (12)

Let us assume, for the sake of contradiction, that there is the smallest value among *t*_*c*_, *t*_*e*_, and *t*_*s*_ delivering non-positive solutions. Consider them sequentially.

Let *t*_*c*_ > 0 be the smallest instance of time such that *n*_*C*_(*t*_*c*_) = 0. From the first and second lines of Eq. 12, we get

$$\begin{array}{ll} & \frac{dn_{S}}{dt}>-k_{1}n_{S}\left(t\right)n_{E}\left(t\right),\\ & \frac{dn_{E}}{dt}>-k_{1}n_{S}\left(t\right)n_{E}\left(t\right). \end{array}$$

It implies that

$$\begin{array}{ll} & n_{S}\left(t\right)>n_{S}\left(0\right)\exp\left(-k_{1}\int_{0}^{t}n_{E}\left(\text{ξ}\right)d\xi \right)>0,\\ & n_{E}\left(t\right)>n_{E}\left(0\right)\exp\left(-k_{1}\int_{0}^{t}n_{E}\left(\xi \right)d\xi \right)>0,\\ & t\in \left[0,t_{c}\right). \end{array}$$

From the third line of Eq. 12, we have

$$\frac{dn_{C}}{dt}>-k_{2}n_{C}\left(t\right)-k_{-1}n_{C},\;t\in \left[0,t_{c}\right).$$

Hence,

$$n_{C}\left(t\right)>n_{C}\left(0\right)\exp\left(-k_{2}-k_{-1}\right)t,\;t\in \left[0,t_{c}\right).$$ (13)

By continuing Eq. 13, we have *n*_*C*_(*t*_*c*_) > 0, which contradicts the initial assumption.

Let *t*_*e*_ be the smallest instant that *n*_*E*_(*t*_*e*_) = 0. From the third part of Eq. 12, it follows that

$$\frac{dn_{C}}{dt}>-k_{2}n_{C}\left(t\right)-k_{-1}n_{C},$$

and *n*_*C*_(*t*) > 0 for *t* ∈ [0, *t*_*e*_).

In turn, from the second part of Eq. 12, we have

$$\frac{dn_{E}}{dt}>-k_{1}n_{S}\left(t\right)n_{E}\left(t\right),$$

and

$$n_{E}\left(t\right)>n_{E}\left(0\right)\exp\left(-k_{1}\int_{0}^{t}n_{S}\left(\xi \right)d\xi \right)>0$$

for *t* ∈ [0, *t*_*e*_],

Let *t*_*s*_ be the smallest instant that *n*_*S*_ (*t*_*s*_) = 0. From the second line of Eq. 12, we get that *n*_*C*_(*t*) > 0, *t* ∈ [0, *t*_*s*_). Furthermore, from the first equation, we have

$$\frac{dn_{S}}{dt}>-k_{1}n_{S}\left(t\right)n_{E}\left(t\right),$$

and

$$n_{S}\left(t\right)>n_{S}\left(0\right)\exp\left(-k_{1}\int_{0}^{t}n_{E}\left(\xi \right)d\xi \right)>0$$

for *t* ∈ [0, *t*_*s*_], which contradicts the assumption.

For the reasons given, we see that the solution of Eq. 12 exists and is positive for any positive initial values (*n*_*S*_(0), *n*_*E*_(0), *n*_*C*_(0), *n*_*P*_(0)) > 0.

##### 3.3.1.2 Two discrete delays

It is natural to assume that the solutions of Eq. 4 are still positive for some sufficiently small *τ*_1_ and *τ*_2_. We aim to offer the conditions of positiveness.

The conditions will be based on the notion of the delayed exponential function. Given the value *x* ∈ **R** and delay *τ* > 0, the delayed exponential function is called (Angstmann et al., 2023)

$$e_{\tau }^{x}≔\overset{+\infty }{\underset{n=0}{\sum }}\frac{{\left(x-n\tau \right)}^{n}}{\Gamma \left(n+1\right)}\Theta \left(\frac{x}{\tau }-n\right),$$

where Θ(⋅) is the Heaviside function. This function has the property that $\frac{d}{dx}e_{\tau }^{\lambda x}=\lambda e_{\tau }^{\lambda \left(x-\tau \right)}$ . Note that contrary to the “undelayed” exponential function, $e_{\tau }^{x}$ can also accept negative values.

To apply the approach of contradictions shown above, let *t*_*c*_, *t*_*e*_, and *t*_*s*_ be instances delivering non-positive solutions.

If *t*_*c*_ > 0 be the smallest instance that *n*_*C*_ (*t*_*c*_) = 0, then from the first and second lines of Eq. 4

$$\begin{array}{ll} & \frac{dn_{S}}{dt}>-k_{1}n_{S}\left(t-\tau_{1}\right)n_{E}\left(t-\tau_{1}\right),\\ & \frac{dn_{E}}{dt}>-k_{1}n_{S}\left(t-\tau_{1}\right)n_{E}\left(t-\tau_{1}\right). \end{array}$$

Hence,

$$\begin{array}{l} n_{S}\left(t\right)>n_{S}\left(0\right)e_{\tau_{1}}^{-k_{1}n_{E_{0}}t},\\ n_{E}\left(t\right)>n_{E_{0}}e_{\tau_{1}}^{-k_{1}n_{S}^{\max}t}, \end{array}$$

where $n_{S}^{\max}≔\max_{u}n_{S}\left(u\right)$ .

From the third line of Eq. 4, we have

$$\frac{dn_{C}}{dt}>-k_{2}n_{C}\left(t-\tau_{2}\right)-k_{-1}n_{C},\;t\in \left[0,t_{c}\right).$$

Consider *t* = *t*_*c*_. Hence,

$$\frac{dn_{C}\left(t_{c}\right)}{dt}>-k_{2}n_{C}\left(t_{c}-\tau_{2}\right),$$

and we see that

$$n_{C}\left(t\right)>n_{C}\left(0\right)e_{\tau_{2}}^{-k_{2}n_{E_{0}}t},t\in \left[0,t_{c}\right].$$

Following the proof in the previous case, we conclude that the solutions of Eq. 4 are positive if rate parameters, initial conditions, and delays *τ*_1_, *τ*_2_ are such that for any *t* > 0

$$e_{\tau_{1}}^{-k_{1}n_{E_{0}}t}>0,e_{\tau_{1}}^{-k_{1}n_{S}^{\max}t}>0,e_{\tau_{2}}^{-k_{2}n_{E_{0}}t}>0.$$

#### 3.3.2 Stability research

##### 3.3.2.1 Equilibrium of the system

Let $\left({\bar{n}}_{S},{\bar{n}}_{E},{\bar{n}}_{C},{\bar{n}}_{P}\right)$ be the equilibrium of Model (4). It should satisfy

$$\begin{array}{ll} & k_{-1}{\bar{n}}_{C}-k_{1}{\bar{n}}_{S}{\bar{n}}_{E}=0,\\ & k_{-1}{\bar{n}}_{C}+k_{2}{\bar{n}}_{C}-k_{1}{\bar{n}}_{S}{\bar{n}}_{E}=0,\\ & k_{1}{\bar{n}}_{S}{\bar{n}}_{E}-k_{2}{\bar{n}}_{C}-k_{-1}{\bar{n}}_{C}=0,\\ & k_{2}{\bar{n}}_{C}=0. \end{array}$$

It follows that

$$\begin{array}{ll} & {\bar{n}}_{C}=0,\\ & k_{1}{\bar{n}}_{S}{\bar{n}}_{E}=0. \end{array}$$

When analyzing Eq. 4, we see that there is a conserved quantity of enzyme because $\frac{d\left(n_{E}+n_{C}\right)}{dt}\equiv 0$ . Hence, $n_{E}\left(t\right)+n_{C}\left(t\right)\equiv n_{E_{0}}$ , where $n_{E_{0}}$ is the total amount of available enzyme. Thus, from the last equality of Section 3.3.2.1, we conclude that ${\bar{n}}_{E}=n_{E_{0}}$ and ${\bar{n}}_{S}=0$ .

So, we have the unique equilibrium of Model (4), which is the substrate and complex free equilibrium (SCFE).

$${\bar{n}}_{S}=0,{\bar{n}}_{E}=n_{E_{0}},{\bar{n}}_{C}=0,{\bar{n}}_{P}=\text{const}.$$

The value of ${\bar{n}}_{P}$ is bounded and can be determined by Eq. 6. Note that it is related to the initial values of both *n*_*S*_ and *n*_*E*_. *n*_*P*_ is not included in the right-hand sides of Eq. 4.

##### 3.3.2.2 Marginal stability

The Jacobian matrix at SCFE for Model (4) is given by

$$J={\left[\begin{array}{cccc} -k_{1}{\bar{n}}_{E}e^{-\lambda \tau_{1}} & -k_{1}{\bar{n}}_{S}e^{-\lambda \tau_{1}} & k_{-1} & 0\\ -k_{1}{\bar{n}}_{E}e^{-\lambda \tau_{1}} & -k_{1}{\bar{n}}_{S}e^{-\lambda \tau_{1}} & k_{-1}+k_{2}e^{-\lambda \tau_{2}} & 0\\ k_{1}{\bar{n}}_{E}e^{-\lambda \tau_{1}} & k_{1}{\bar{n}}_{S}e^{-\lambda \tau_{1}} & -k_{2}e^{-\lambda \tau_{2}}-k_{1} & 0\\ 0 & 0 & k_{2}e^{-\lambda \tau_{2}} & 0 \end{array}\right]}_{\text{SCFE}}=\left[\begin{array}{cccc} -k_{1}n_{E_{0}}e^{-\lambda \tau_{1}} & 0 & k_{-1} & 0\\ -k_{1}n_{E_{0}}e^{-\lambda \tau_{1}} & 0 & k_{-1}+k_{2}e^{-\lambda \tau_{2}} & 0\\ k_{1}n_{E_{0}}e^{-\lambda \tau_{1}} & 0 & -k_{2}e^{-\lambda \tau_{2}}-k_{1} & 0\\ 0 & 0 & k_{2}e^{-\lambda \tau_{2}} & 0 \end{array}\right].$$

The characteristic quasi-polynomial for $\left({\bar{n}}_{S},{\bar{n}}_{E},{\bar{n}}_{C},{\bar{n}}_{P}\right)$ is given by

$$\chi \left(\lambda \right)=\lambda^{4}+k_{-1}\lambda^{3}+k_{1}\left({\bar{n}}_{S}+{\bar{n}}_{E}\right)\lambda^{3}e^{-\lambda \tau_{1}}+k_{2}\lambda^{3}e^{-\lambda \tau_{2}}+k_{1}k_{2}{\bar{n}}_{E}\lambda^{2}e^{-\lambda \left(\tau_{1}+\tau_{2}\right)}=\lambda^{2}\chi_{1}\left(\lambda \right),$$

where

$$\chi_{1}\left(\lambda \right)=\lambda^{2}+k_{-1}\lambda +k_{1}\left({\bar{n}}_{S}+{\bar{n}}_{E}\right)\lambda e^{-\lambda \tau_{1}}+k_{2}\lambda e^{-\lambda \tau_{2}}+k_{1}k_{2}{\bar{n}}_{E}e^{-\lambda \left(\tau_{1}+\tau_{2}\right)}.$$

Hence, stability analysis can be reduced to obtaining conditions of non-positive values of real parts of the quasi-polynomial of *χ*_1_(*λ*).

For SCFE, we have the following quasi-polynomial:

$$\chi_{1}\left(\lambda \right)=\lambda^{2}+k_{-1}\lambda +k_{1}n_{E_{0}}\lambda e^{-\lambda \tau_{1}}+k_{2}\lambda e^{-\lambda \tau_{2}}+k_{1}k_{2}{\bar{n}}_{E}e^{-\lambda \left(\tau_{1}+\tau_{2}\right)}.$$

When applying the Padé approximation shown in Eq. 11 to the characteristic quasi-polynomial, we get

$$\begin{array}{ll} \chi_{1}\left(\lambda \right)\approx & \left(k_{1}{\bar{n}}_{E}\lambda^{3}\tau_{1}^{2}k_{2}\tau_{2}-2k_{1}{\bar{n}}_{E}\lambda^{3}\tau 1^{2}-k_{1}{\bar{n}}_{E}\lambda^{4}\tau_{1}^{2}\tau_{2}+-2k_{1}{\bar{n}}_{E}\lambda^{2}\tau 1^{2}k_{2}+4k_{1}{\bar{n}}_{E}\lambda^{2}\tau_{2}\right.\\ & -4k_{1}{\bar{n}}_{E}\lambda k_{2}\tau_{2}+8k_{1}{\bar{n}}_{E}\lambda +8k_{1}{\bar{n}}_{E}k_{2}+\lambda^{5}\tau_{1}^{2}\tau_{2}+\lambda^{4}\tau 1^{2}k_{-1}\tau_{2}+2\lambda^{4}\tau_{1}^{2}\\ & -\lambda^{4}\tau_{1}^{2}k_{2}\tau_{2}+4\lambda^{4}\tau_{1}\tau_{2}+2\lambda^{3}\tau_{1}^{2}k_{-1}+2\lambda^{3}\tau_{1}^{2}k_{2}+4\lambda^{3}\tau_{1}k_{-1}\tau_{2}-4\lambda^{3}\tau_{1}k_{2}\tau_{2}\\ & \left.+8\lambda^{3}\tau_{1}+4\lambda^{3}\tau_{2}+8\lambda^{2}\tau_{1}k_{-1}+8\lambda^{2}\tau_{1}k_{2}+4\lambda^{2}k_{-1}\tau_{2}-4\lambda^{2}k_{2}\tau_{2}+8\lambda^{2}+8\lambda k_{-1}+8\lambda k_{2}\right)/\left(\lambda^{3}\tau_{1}^{2}\tau_{2}+2\lambda^{2}\tau_{1}^{2}+4\lambda^{2}\tau_{1}\tau_{2}+8\lambda \tau_{1}+4\lambda \tau_{2}+8\right). \end{array}$$

Setting the values for ${\bar{n}}_{E}=1.139071mM$ , *k*_1_, *k*_2_, *k*_−1_ from Π_*opt*_ (Table 2), we obtain

$$\chi_{1}\left(\lambda \right)=0.0992985372e^{-\lambda \tau_{1}}\lambda +0.0121224945e^{-\lambda \tau_{1}}e^{-\lambda \tau_{2}}+\lambda^{2}+0.1220813\lambda e^{-\lambda \tau_{2}}+0.1048326\lambda .$$

In turn, applying the Padé approximation yields

$$\begin{array}{ll} \chi_{1}\left(\lambda \right)\approx & \left(8\left(\left(\lambda^{5}\tau_{1}^{2}\tau_{2}\right)/8+\left(-0.9323778976\lambda^{4}\tau_{1}^{2}\tau_{2}\right)/64+\left(\lambda^{4}\tau_{1}^{2}\right)/4+\left(\lambda^{4}\tau_{1}\tau_{2}\right)/2\right.\right.+\left(0.1939599121\lambda^{3}\tau_{1}^{2}\tau_{2}\right)/128+\left(0.2552307255\lambda^{3}\tau_{1}^{2}\right)/8+\left(-4.4156672\lambda^{3}\tau_{1}\tau_{2}\right)/512\\ & +\lambda^{3}\tau_{1}+\left(\lambda^{3}\tau_{2}\right)/2+\left(-0.048489978\lambda^{2}\tau_{1}^{2}\right)/16+\left(1.8153112\lambda^{2}\tau_{1}\right)/8+\left(0.3281993488\lambda^{2}\tau_{2}\right)/8\left.\left.+\lambda^{2}+\left(-0.048489978\lambda \tau_{2}\right)/8+0.3262124372\lambda +0.0121224945\right)\right)/\left(\lambda^{3}\tau_{1}^{2}\tau_{2}+2\lambda^{2}\tau_{1}^{2}\right.\\ & \left.+4\lambda^{2}\tau_{1}\tau_{2}+8\lambda \tau_{1}+4\lambda \tau_{2}+8\right). \end{array}$$

Consider two special cases of Eq. 4:

**Case 1** without delays, that is, *τ*_1_ = *τ*_2_ = 0. Then,

$$\chi_{1}\left(\lambda \right)=\lambda^{2}+0.3262124372\lambda +0.0121224945,$$

and we have the roots of *χ*_1_(*λ*):

$$\lambda_{1}=-0.28344384,\;\lambda_{2}=-0.04276859,$$

which means marginal stability in this case.

**Case 2** with optimal values of the delays from Π_*opt*_ (Table 2), that is, *τ*_1_ = 15.69849 and *τ*_2_ = 5.357829. It holds

$$\begin{array}{ll} \chi_{1}\left(\lambda \right)= & 0.0992985372e^{-15.69849\lambda }\lambda +0.0121224945e^{-15.69849\lambda }e^{-5.357829\lambda }\\ & +\lambda^{2}+0.1220813\lambda e^{-5.357829\lambda }+0.1048326\lambda . \end{array}$$

#### 3.3.3 Effect of delays on qualitative behavior

We investigated the qualitative behavior of the model by selecting specific parameter values, denoted as Π_*opt*_, and examining the influence of the delay *τ*_1_ on Model 4’s dynamics, provided that *τ*_2_ = 5.357829 is fixed. To explore this influence, we varied the parameter *τ*_1_ over the interval [0, 35].

**FIGURE 7:**
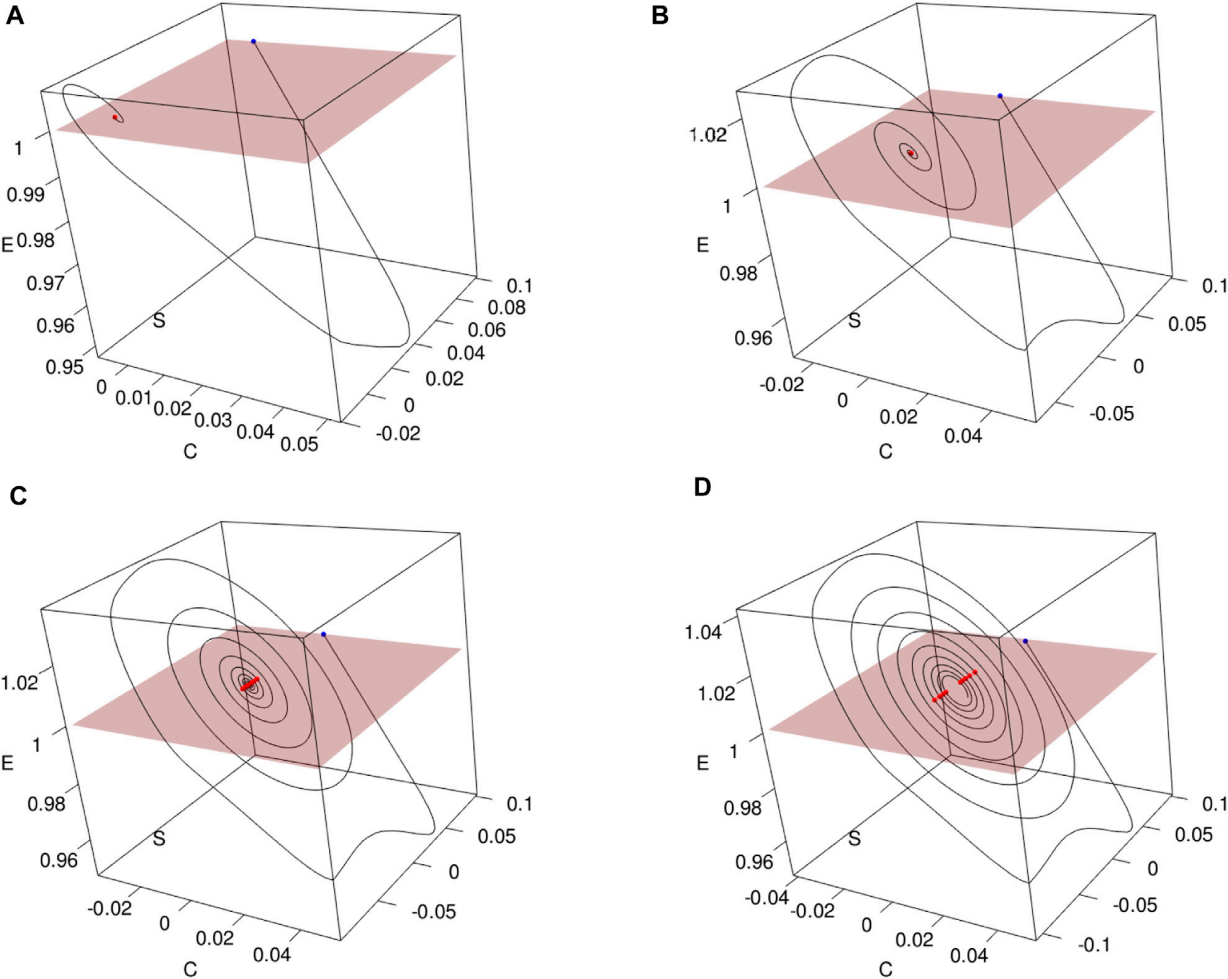
Constructing Poincaré sections based on trajectories (*n*_*S*_(*t*), *n*_*E*_(*t*), *n*_*C*_(*t*)) of Model (4) with the parameters Π_*opt*_ in dependence of **(A)** *τ*_1_ =15.69849, **(B)** *τ*_1_ = 25, **(C)** *τ*_1_ = 32, and **(D)** *τ*_1_ = 35. Poincaré sections (in red) are obtained as a result of crossing with the plane *n*_*E*_ = *d*, where *d* =(min_*t*_ *n*_*E*_(*t*) + max_*t*_ *n*_*E*_(*t*))/2.

Figure 7 illustrates the construction of Poincaré sections in 3D plots for various values of *τ*_1_. These sections provide insights into the system’s behavior at specific points in its phase space. As it can be seen for the values of *τ*_1_ and *τ*_2_ greater than in Π_*opt*_, we lose the positivity of the solutions. This phenomenon was evidenced in Section 3.3.1.2, where it was shown that in contrast to the model without delay, positivity requires restricting the parameters in Eq. (4).

**FIGURE 8:**
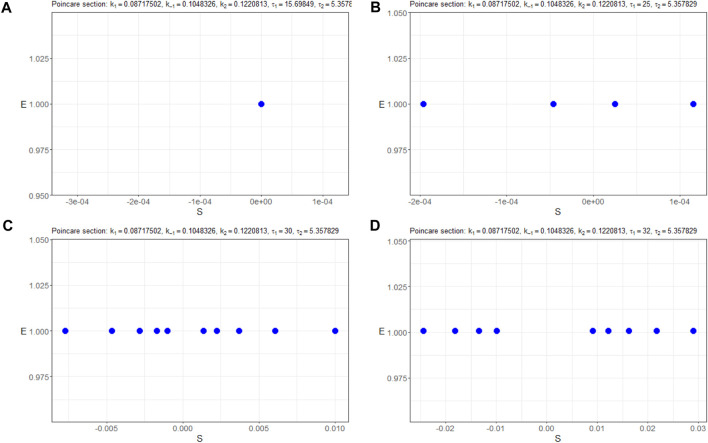
Poincaré sections (in blue) in the (*n*_*S*_, *n*_*E*_)-plane of the model (4) with the parameters Π_*opt*_ in dependence of **(A)** *τ*_1_ = 15.69849, **(B)** *τ*_1_ = 25, **(C)** *τ*_1_ = 32, and **(D)** *τ*_1_ = 35.

Furthermore, Figure 8 presents the corresponding Poincaré sections plotted in the (*n*_*S*_, *n*_*E*_)-plane, offering a clearer visualization of the system’s trajectory crossings. A thorough inspection of the sections says that for the initial trajectory (*n*_*S*_(*t*), *n*_*E*_(*t*), *n*_*C*_(*t*)) behaves at the equilibrium state as a stable node. Then, at less than *τ* ≈ 25, Hopf bifurcation appears, and the limit cycle starts from a small radius of approximately 10^–4^ and is extended sequentially to 10^–2^. Simultaneously, period doubling occurs.

**FIGURE 9:**
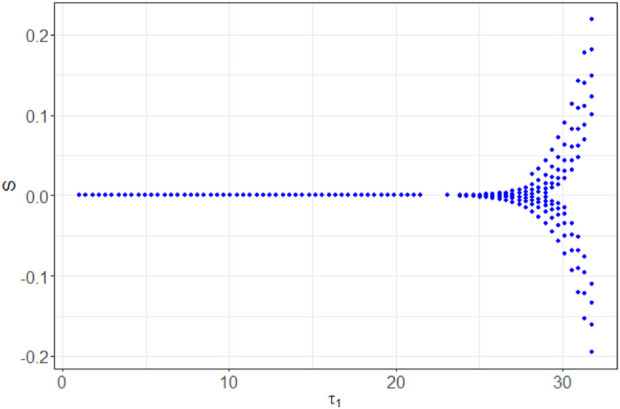
Bifurcation diagram of *n*_*S*_ with respect to the parameter *τ*_1_.

**FIGURE 10:**
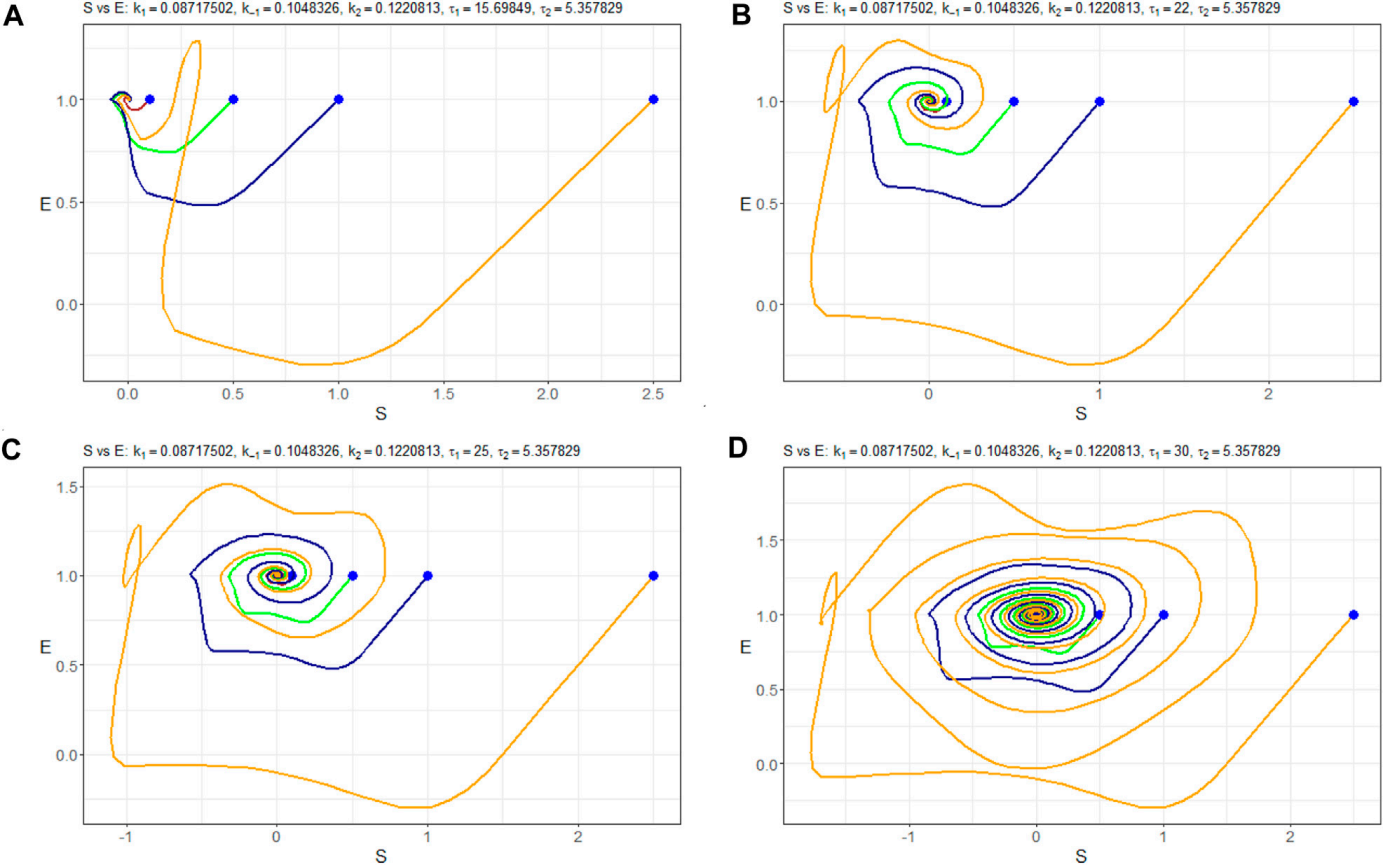
(*n*_*S*_, *n*_*E*_)-phase plots of Model (4) with the parameters Π_*opt*_ in dependence of **(A)** *τ*_1_=15.69849, **(B)** *τ*_1_=25, **(C)** *τ*_1_=32, and **(D)** *τ*_1_=35. Trajectories are constructed at different initial values of *n*_*S*_ (0):–0.1 mM, –0.5 mM, –1.0 mM, and –2.5 mM. Starting points are indicated in blue.

In Figure 9, we depict the bifurcation diagram for the variable *n*_*S*_ resulting from the parameter variation of *τ*_1_. It reveals that the equilibrium state behaves as a stable node within the range of *τ*_1_ values from 0 to 25. Subsequently, a bifurcation occurs, leading to a transition to a limit cycle. This period-doubling phenomenon is further supported by Figure 10, which displays the (*n*_*S*_, *n*_*E*_)-phase plane for different initial states.

**FIGURE 11:**
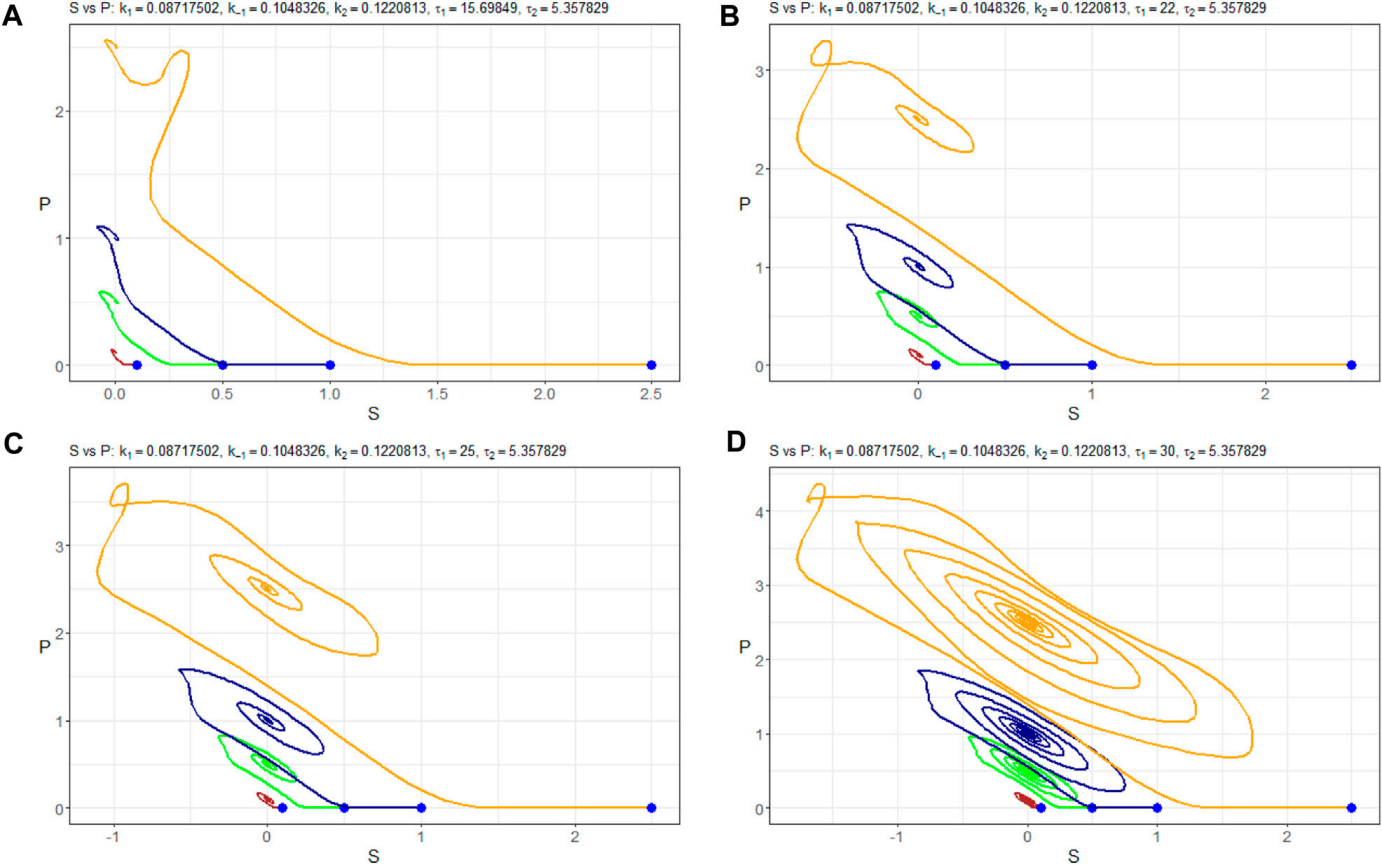
(*n*_*S*_, *n*_*P*_)-phase plots of Model (4) with the parameters Π_*opt*_ in dependence of **(A)** *τ*_1_=15.69849, **(B)** *τ*_1_=25, **(C)** *τ*_1_=32, and **(D)** *τ*_1_=35. Trajectories are constructed at different initial values of *n*_*S*_ (0):–0.1 mM, –0.5 mM, –1.0 mM, and –2.5 mM. Starting points are indicated in blue.

For *τ*_1_ values exceeding 32, the system’s behavior appears likely to become chaotic. This transition is occurring through period doubling, although further investigation is warranted. It is noteworthy that the system exhibits marginal stability, as evident from Figure 11. This figure illustrates how different initial values of *n*_*S*_ (0) lead the solution *n*_*P*_ to converge toward either a stable node or a limited cycle.

Special attention should be paid to the positivity of the solutions, as we observed from the numerical modeling.

## 4 Discussion

The use of gamma-distributed delays and discretely distributed delay models is simultaneously important because the parameter values from the IR1CB model can be used for the IR1C2DMM model. The gamma-distributed delays model allows us to estimate the mean value of the delay easily.

Moreover, based on the results of numerical experiments, we can conclude that for the optimal values of the parameters Π_*opt*_ (Tables 1, 2), we have that *τ*_1_ + *τ*_2_ ≈ E(*τ*), where *τ*_1_, *τ*_2_ are the discrete delays from Eq. 4, and *τ* is the distributed delay from Eq. 2 (see also Figure 4 for delay density distribution).

The use of the Levenberg–Marquardt algorithm is essentially determined by choosing the initial approximation of the parameter values. It can be improved by applying AI techniques and constructing and tuning neural networks of the appropriate architecture for future consideration.

Artificial intelligence (AI) models like recurrent NNs can also be used to model enzyme–substrate interaction (we will try this in the future). The advantage of an AI model is that it makes more accurate predictions. The disadvantages of using AI are (1) the black box problem, meaning that the models are not based on any biochemical assumptions but only neural network architecture and (2) the overfitting problem, meaning that when we try to fit the outputs as accurately as we can, we may not see the general tendencies in reactions.

The system is characterized by marginal stability, which is between Lyapunov stability and instability. It corresponds to the objective of an electrochemical biosensor as a measuring device. Each initial state, including substrate concentration changes, has its “own” concentration of the product at an infinite time. We can reformulate it as the definition of operating stability.

The question arises of how to apply and interpret the marginal stability condition. As was shown, the marginal stability condition can be reduced by checking the real parts of the polynomial roots. We see that the roots depend on the reaction rate parameters (*k*_1_, *k*_2_, *k*_−1_), time delays *τ*_1_, *τ*_2_, and the initial concentration of $n_{E_{0}}$ .

## 5 Conclusion

The equilibrium of the system was demonstrated in this article. From the viewpoint of enzymatic reactions and based on the proposed model, we saw that the system would be in equilibrium in the case of the absence of substrate and complex, that is, ${\bar{n}}_{S}\equiv 0$ , *n*_*C*_ ≡ 0, as well as the concentration of the enzyme equal to the total amount of available enzyme. The concentration of the product should be a constant value calculated in accordance with Eq. 6 as the area under curve *n*_*C*_(*t*), that is, ${\bar{n}}_{P}\equiv const$ . In addition, ${\bar{n}}_{P}$ is determined by the initial value *n*_*S*_ (0).

In the work, we also considered a model with two discrete delays where *τ*_1_ and *τ*_2_ are time durations required for the entire fulfillment of forwarded reactions. To be precise, *τ*_1_ is the time needed for the substrate to bind with the enzyme (time needed for complex formation), and *τ*_2_ is the time needed for the complex to break down into enzyme and product. It was numerically shown that increasing those two parameters could result in a loss of stability. Suppose we have optimal values $\tau_{1}^{0}$ and $\tau_{2}^{0}$ and they correspond to a stable solution. If $\tau_{1}>\tau_{1}^{0}$ , more time is required for forming the complex at $\text{S}+\text{E}\overset{{\text{k}}_{1}\text{,}\tau_{1}}{\to }\text{C}$ . In other words, less complex will be produced compared with the optimal value. In turn, if $\tau_{2}>\tau_{2}^{0}$ , this means that more time will be needed for the $\text{C}\overset{{\text{k}}_{1}\text{,}\tau_{2}}{\to }\text{E}+\text{P}$ reaction, leading to less enzyme and product than in the steady state. We remember that in the steady state, it has been proven that we have no complex, and the enzyme content is maximal. On the other hand, an increase in *τ*_2_ results in having more complex but less product. This will affect the final content of the product, that is, the biosensor index.

It can also be noted that no unique parameters are set for fitting the model to the expected data at different values (Figures 5, 6), as the rates are not constant but can be functions of the substance concentrations. In particular, the times required for the reactions $\text{S}+\text{E}\overset{{\text{k}}_{1}\text{,}\tau_{1}}{\to }\text{C}$ and $\text{C}\overset{{\text{k}}_{1}\text{,}\tau_{2}}{\to }\text{E}+\text{P}$ are not constant. They likely depend on the concentrations of S, E (for *τ*_1_), and C (for *τ*_2_). These data should be determined experimentally. Moreover, the models can be extended by involving other complexes created by reactions with other biosensor auxiliary components. For example, BSA is known to create a complex with an enzyme known as CLEA (Shah et al., 2006). In other words, incorporating additional variables and increasing the system dimension can improve the model fitting.

These findings provide valuable insights into the system’s dynamics and highlight the importance of parameter exploration in understanding the qualitative behavior of enzyme kinetics models.

## Data Availability

The raw data supporting the conclusion of this article will be made available by the authors, without undue reservation.

## Footnotes

1 Without loss of generality, we assume that all time series have the same time span.
